# Endothelium‐Independent Relaxation of Alkaloid Boldine in Isolated Aortas From Normotensive and Hypertensive Rats: Participation of Ca^2+^ Channels

**DOI:** 10.1002/cbdv.202501752

**Published:** 2025-08-25

**Authors:** Martina Odebrecht Cavichiolo, Rita de Cássia Vilhena da Silva, Valdir Cechinel Filho, Priscila de Souza

**Affiliations:** ^1^ Postgraduate Program in Pharmaceutical Sciences Universidade Do Vale Do Itajaí Itajaí Santa Catarina Brazil

**Keywords:** alkaloid, aorta, calcium channels, vascular tone

## Abstract

The regulation of vascular tone plays a fundamental role in blood pressure homeostasis and still represents a significant challenge in clinical practice. Boldine, a naturally occurring alkaloid from *Peumus boldus*, has emerged as a compound of interest due to its therapeutic potential. This study explored boldine's vasorelaxant effects on aortas of normotensive (NTR) and spontaneously hypertensive (SHR) rats. *N*ω‐Nitro‐l‐arginine methyl ester (l‐NAME) (a nitric oxide synthase inhibitor), ODQ (a soluble guanylate cyclase inhibitor), and tetraethylammonium (TEA) (a nonselective K⁺ channel blocker) exerted a modest inhibitory effect, causing a rightward shift in the boldine‐induced relaxation curves without significantly affecting the maximal relaxation. Boldine affected both endoplasmic reticulum‐based IP_3_ and ryanodine receptors (RyRs) and transmembrane calcium channels in NTR aortas, whereas in SHR aortas, its actions were linked to IP_3_ and RyRs receptors. These findings indicate that boldine's vasorelaxant effects may contribute to vascular tone regulation through modulation of Ca^2^⁺ channels.

## Graphical abstract

Involvement of Ca^2+^ channels in boldine‐induced vascular relaxation.

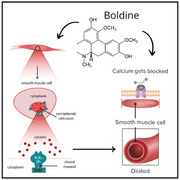

## 1 Introduction

Cardiovascular disease (CVD) is a comprehensive term that refers to various pathologies affecting the cardiovascular system, such as coronary artery disease, myocardial infarction, stroke, atherosclerosis, cardiomyopathy, and others [1]. CVDs are the leading cause of morbidity and mortality worldwide, and their global burden is continuously increasing in many countries, as reported by the Global Burden of Disease study [2]. Lifestyle factors such as an unbalanced diet, alcohol consumption, smoking, physical inactivity, and aging are risk factors for the occurrence and progression of CVD [3].

Systemic arterial hypertension (SAH) is the most significant reversible risk factor for CVD. As it is often asymptomatic, SAH tends to progress with structural and/or functional changes in target organs, such as the heart, brain, kidneys, and vessels [4]. Globally, around 1.3 billion adults are affected due to hypertension. High systolic blood pressure (≥110–115 mmHg) was the single most important risk factor for early death worldwide, leading to an estimated 10.8 million avoidable deaths every year [2].

Commercially available antihypertensive drugs act through various mechanisms, but their effectiveness is often limited by side effects—such as fatigue (β‐blockers), peripheral edema and flushing (calcium channel blockers), dizziness or hyperkalemia (angiotensin receptor blockers), and electrolyte imbalances (diuretics)—which can significantly reduce patient adherence to treatment [5, 6]. Therefore, the search for new therapeutic options that facilitate adherence to treatment is becoming increasingly necessary. In this context, medicinal plants represent an extensive and renewable resource, whether for use in their natural form or for the discovery of extracts with therapeutic potential for the formulation of new drugs [7].

It is well known that plants contain hundreds of secondary metabolites. Therefore, the search for these metabolites becomes important for expanding the therapeutic arsenal, increasing efficiency, and promoting greater adherence to pharmacological treatment. Considering this, the alkaloid boldine, (*S*)‐2,9‐dihydroxy‐1,10‐dimethoxyaporphine, found abundantly in the leaves and bark of boldo (*Peumus boldus* Molina), stands out. It has been the subject of various scientific studies supporting the current proposal, including its renal protective effect in animal models of diabetes [8] and hypertension [9], diuretic effect [10], beneficial effects on endothelial dysfunction [11, 12, 13], and anti‐inflammatory effects in different experimental models [14, 15].

Despite the wealth of scientific evidence of boldine, there are still many gaps to be explored to better understand its therapeutic benefits. Hence, leveraging the previously described properties of boldine, this research aimed to examine its vascular effects in both normotensive (NTR) and spontaneously hypertensive (SHR) rats. Understanding its vasorelaxant potential, as well as the molecular mechanisms involved, may lead to new therapeutic strategies for the treatment of CVDs, through the modulation of vascular tone, which is one of the essential pillars for adequate blood pressure control.

## 2 Materials and Methods

### 2.1 Animals

Male NTR and SHR Wistar rats aged 3–4 months were used, provided by the central vivarium of the Universidade do Vale de Itajaí (UNIVALI). The animals were maintained at controlled room temperature (22°C ± 2°C), 12‐h light/dark cycle, and relative humidity of 50% + 5%, with free access to water and food.

### 2.2 Drugs and Salts

The following drugs and salts were used to conduct the experimental protocols: phenylephrine (Phe); acetylcholine (ACh); *N*ω‐nitro‐l‐arginine methyl ester (l‐NAME); 1H[1,2,4]oxadiazol[4,3‐a]quinoxalin‐1‐one (ODQ); indomethacin (Ind); glibenclamide (GLB); tetraethylammonium (TEA); 4‐aminopyridine (4‐AP); atropine (ATR); propranolol (PROP); barium chloride (BaCl_2_); caffeine; nifedipine (NIFE); and boldine (purity ≥98%), which were all purchased from Sigma‐Aldrich Corporation (St. Louis, Missouri, USA). For the preparation of physiological saline solution (PSS—mM): sodium chloride (NaCl); calcium chloride (CaCl_2_); potassium chloride (KCl); magnesium sulfate (MgSO_4_); potassium dihydrogen phosphate (KH_2_PO_4_); sodium bicarbonate (NaHCO_3_); d‐glucose; and ethylenediaminetetraacetic acid (EDTA) purchased from the company MERCK (Darmstadt, Germany). Xylazine and ketamine were purchased from VETEC (Duque de Caxias, RJ, Brazil).

### 2.3 Determination of Vascular Reactivity in a Rat‐Isolated Aorta Model

Vascular reactivity was assessed in isolated rat aortic rings, following previously described protocols [16]. The animals were housed under standard laboratory conditions until the day of the experiment. For thoracic aorta extraction, animals were anesthetized with an intraperitoneal injection of ketamine (80 mg/kg) and xylazine (10 mg/kg). Once deep anesthesia was achieved, a thoracotomy was performed, and the descending thoracic aorta was excised and transferred to a petri dish containing pre‐warmed PSS to remove the connective tissue. The aorta was then sectioned into rings approximately five millimeters in length. These rings were placed in an isolated organ bath system with 2 mL glass chambers containing PSS (composition in mM: NaCl 110.8, KCl 5.9, NaHCO_3_ 25, MgSO_4_ 1.07, CaCl_2_ 2.49, KH_2_PO_4_ 2.33, and glucose 11.51), constantly aerated with carbogen (95% O_2_/5% CO_2_) and maintained at 37°C. Each ring was subjected to a basal tension of 1 g and mounted between two metal rods—one fixed and the other connected to an isometric transducer. The transducer was linked to a signal amplifier (DATAQ Instruments) and connected to a computer with specialized integration software (WinDaq Software, DATAQ Instruments, Akron, Ohio, USA) for recording.

A stabilization period of 1 h with PSS changes every 15 min was adopted. After this period, tissue responsiveness was tested with the addition of 60 mM KCl. Following a 30‐min stabilization period, contraction was induced with Phe 1 µM, followed by Ach 1 µM during the tonic phase of contraction. ACh‐induced relaxation greater than 80% was used as a criterion to confirm endothelial integrity. This threshold is commonly used as a functional indicator of intact endothelium, as ACh‐mediated vasodilation is primarily endothelium‐dependent and involves nitric oxide (NO) release from endothelial cells [17]. In experiments with endothelium‐denuded aortas, the endothelium was carefully removed using a metal rod. After confirming the integrity of the endothelium, the aortic rings were washed three times with PSS. An additional stabilization period of 30 min was adopted, after which the rings were incubated with boldine at concentrations of 10, 30, and 100 µM for 30 min, constantly aerated with carbogen (95% O_2_/5% CO_2_), and maintained at 37°C. Subsequently, they were exposed to cumulative increasing concentrations of Phe (1 nM–0.3 mM) to evaluate vasoconstrictor effects. The results were expressed in grams.

In another experimental set, after the stabilization of the aortic rings and the confirmation of their responsiveness and endothelial integrity—or following manual removal—the rings were exposed to cumulative increasing concentrations of the boldine compound (1 nM–0.3 mM) to investigate its potential vasodilatory effect against the previously induced contraction with Phe (1 µM). The results obtained were compared between the groups and expressed as a percentage of relaxation.

### 2.4 Role of NO and Prostanoids in the Vasorelaxant Effect of Boldine

Following the assessment of the viability of the aortic ring described in the previous section, a subsequent 30‐min stabilization period was instituted, with PSS renewal on each 15 min. The aortic rings were then incubated with the following pharmacological agents: l‐NAME (10 µM), a nonselective inhibitor of nitric oxide synthase (NOS) enzymes; ODQ (10 µM), a selective inhibitor of soluble guanylate cyclase (sGC); or Ind (10 µM), a nonselective inhibitor of cyclooxygenase enzyme, for 30 min. In the presence of these substances (each in a separate vessel), a contraction was elicited using Phe, and, in the tonic phase of this contraction, boldine was added at cumulative concentrations of 1 nM–0.3 mM. The resultant effects of boldine, both in the presence and absence of these inhibitors, were then compared and quantified as a percentage of the relaxation.

### 2.5 Assessment of the Involvement of Muscarinic and β‐Adrenergic Receptors in the Vasorelaxant Effect of Boldine

After confirmation of aortic viability and a 30‐min wait for stabilization, different preparations were incubated for a period of 30‐min with ATRO (1 µM), a nonselective antagonist of muscarinic receptors, or PROP (10 µM), a nonselective blocker of adrenergic receptors. In the presence of these substances (one in each bath), a contraction was induced by Phe, and, during the tonic phase of this contraction, boldine was added in cumulative concentrations from 1 nM to 0.3 mM. The effects of the compound in the presence and absence of these blockers were compared and expressed as a percentage of the relaxation.

### 2.6 Evaluation of the Involvement of K^+^ Channels in the Vasorelaxant Effect of Boldine

After verifying viability and stabilization, the aortic rings were incubated with the following K^+^ channel blockers: TEA (10 and 1 mM), a nonselective blocker of K^+^ channels subgroups 2.1, 2.2, and 2.3 and a blocker of Ca^2+^‐activated K^+^ channels subgroups 2.3 and 3.1, respectively; or GLB (10 µM), a selective K_ir_ 6.1 and 6.2 blocker (also known as ATP‐sensitive inward rectifier potassium channels); 4‐AP (1 mM), a selective voltage‐gated K^+^ channel blocker; or BaCl_2_ (10 µM), a nonselective influx‐rectifying K^+^ channel blocker. In the presence of these substances (one in each bath), after 30 min, a contraction was induced by Phe, and, during the tonic phase of this contraction, boldine was added in cumulative concentrations from 1 nM to 0.3 mM. The effects of the compounds in the presence and absence of these blockers were compared.

### 2.7 Involvement of Extra‐ and Intracellular Ca^2+^ Channels in the Vasorelaxant Effect of Boldine

Following viability and stabilization checks, to evaluate the role of extracellular Ca^2+^ channels, the PSS was substituted with a calcium‐free depolarizing PSS (KCl—60 mM), followed by a 30‐min wait for stabilization. Different concentrations of boldine (1, 3, and 10 µM) were incubated, and after 30 min, a concentration–response curve (CRC) was constructed with CaCl_2_ solution (0.3 µM–1 M) in each bath. A control setup without boldine and a positive control setup with the addition of 1 µM NIFE, a voltage‐dependent calcium channel blocker, were employed for comparison purposes.

To assess the role of intracellular calcium in the vasorelaxant effect of boldine, after a 30‐min stabilization period, the preparations were washed with calcium‐free PSS. In the first minute following the switch, four to five washes with the calcium‐free solution were performed. After washing, a 15‐min wait for stabilization ensued, and then the rings were exposed to different concentrations of boldine (1, 3, and 10 µM). Thirty minutes after exposure to these concentrations, a new contraction was induced by either Phe (1 µM) or caffeine (100 mM). The controls used were the same as described above. The results obtained were compared between groups.

### 2.8 Statistical Analysis

The results were expressed as the mean ± standard error of the mean (*n* = 6–8 animals per group). One‐ or two‐way analysis of variance (ANOVA) followed by the Bonferroni test or Student's *t*‐test, as applicable, was performed. A *p* value of less than 0.05 was considered statistically significant. Analyses were conducted using GraphPad Prism version 8.00 for Windows (GraphPad Software, La Jolla, CA, USA).

## 3 Results and Discussion

Vascular dysfunction has been identified as a central pathophysiological mechanism in a variety of conditions, including arterial hypertension, atherosclerosis, and diabetes. In its resting state, the arterial bed exhibits a baseline condition of vasoconstriction, known as vascular tone, which is finely regulated by a complex interplay among central control systems, such as the sympathetic nervous system, peripheral systems like the renin–angiotensin–aldosterone system, and local mechanisms, including the endothelium. Dysfunctions in these regulatory pathways can lead to a loss of adequate control of vascular tone, resulting in impairment of the overall functioning of the cardiovascular system [17]. In this manner, the direct effects of boldine on systems involved in blood pressure regulation were evaluated as a way of filling existing gaps regarding its biological effects already described. The methodology involved ex vivo assessment of vascular reactivity using an isolated organ bath system with thoracic aorta rings from NTR and SHR male rats. SHRs are among the most commonly used models for research involving cardiovascular disorders, as they develop hypertension spontaneously [18]. Its importance is attributed to the similarity of its pathophysiology to primary (essential) hypertension in humans [19]. However, females, due to hormonal protection, do not develop significant hypertension, being classified only as prehypertensive. For this reason, male rats are used for research for this purpose.

**FIGURE 1:**
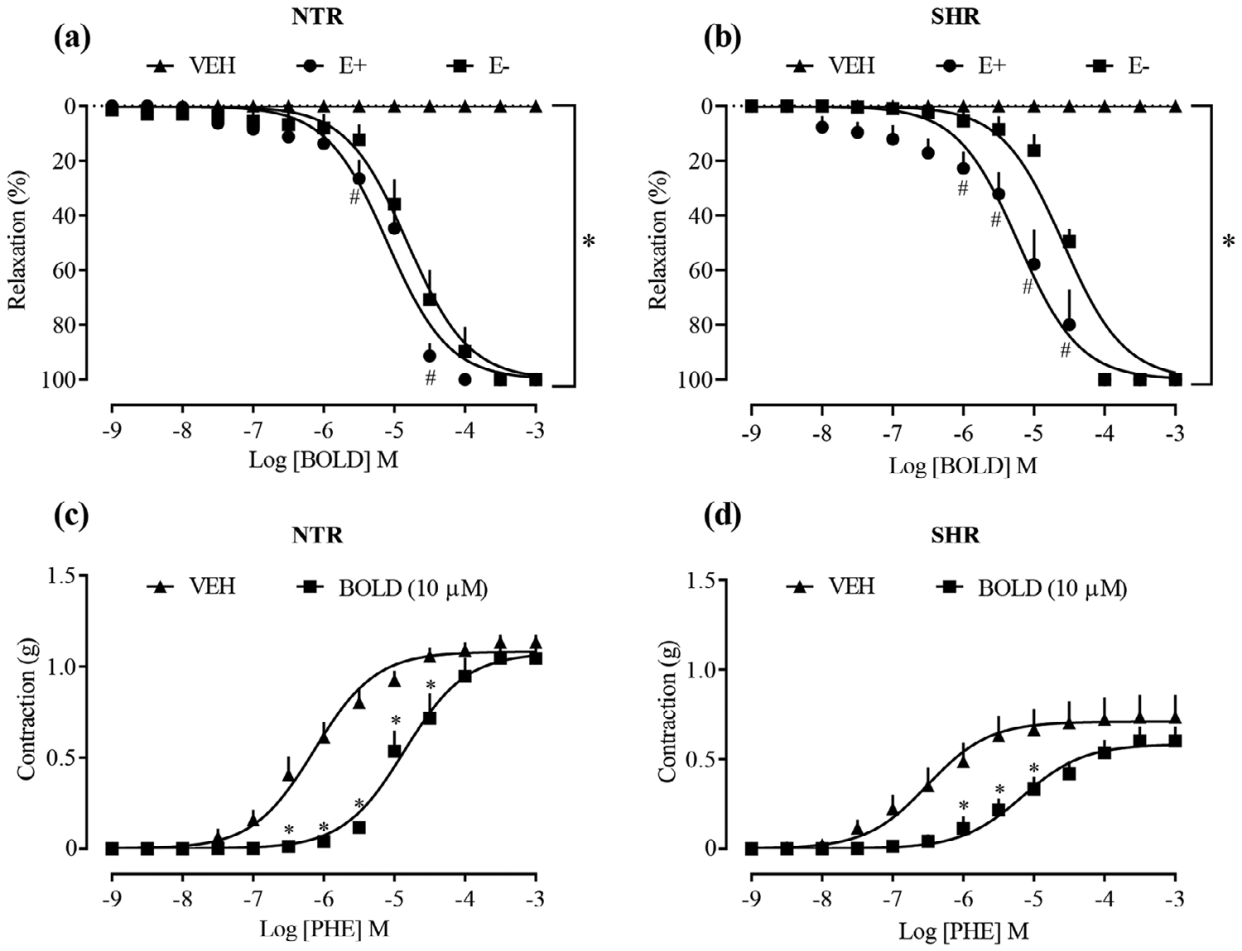
Effect of boldine on vascular reactivity in aortic rings of NTR and SHR. Relaxation of boldine in aortic rings from NTR (a) and SHR (b). E+ and E− indicate endothelium‐intact and endothelium‐denuded preparations, respectively. Inhibition of vascular contraction induced by phenylephrine (Phe) by boldine in aortic rings from NTR (c) and SHR (d). Statistical analyses were performed using a two‐way analysis of variance followed by Bonferroni's multiple comparison test. ∗*p *< 0.05 when compared to the vehicle group and #*p *< 0.05 when compared to the endothelium‐intact and endothelium‐denuded groups. NTR, normotensive rat; SHR, spontaneously hypertensive rat.

The first experiment aimed to evaluate the vasorelaxant effect from cumulative concentrations of boldine (1 nM–0.3 mM), which promoted 100% relaxation in the aortic rings of male NTR (Figure 1a) and SHR (Figure 1b) with and without endothelium, previously contracted with Phe. LogEC50 −5.097 (NTR/E+)/LogEC50 −4.814 (NTR/E) and LogEC50 −5.221 (SHR/E+)/Log EC50 −4.589 (SHR/E−). In addition, exposure of the aortic rings to boldine concentration (10 µM) reduced the contraction induced by Phe in both NTR and SHR groups (Figure 1c,d, respectively). The aortas of the SHR vehicle group showed hypocontractility to Phe (0.527 ± 0.066 g) when compared to the NTR vehicle group (1.205 ± 0.072 g). Although SHRs are commonly associated with hypercontractile responses, in some experimental settings, a reduced sensitivity to vasoconstrictors may occur, as observed in the present study. This hyporesponsiveness could be attributed to alterations in α₁‐adrenergic receptor density or signaling, endothelial dysfunction, or compensatory mechanisms due to chronic sympathetic overstimulation. These factors may modulate vascular tone and reduce the responsiveness to vasoconstrictors. However, an alternative explanation—proposed decades ago—is that the apparent hyporesponsiveness of SHR blood vessels may result from their elevated basal myogenic tone, likely due to increased calcium influx into vascular smooth muscle, which masks the true responsiveness to vasoactive agents [20].

As seen, CVDs develop due to alterations in organic structures, particularly in the vessels and arteries, where endothelial dysfunction occurs, subsequently impairing the function of several mechanisms that rely on endothelial integrity [17]. The endothelium plays a crucial role in modulating vascular tone by synthesizing and releasing a variety of endothelium‐derived contracting factors and endothelium‐derived relaxing factors (EDRF), including vasodilatory prostaglandins, endothelium‐dependent hyperpolarizing factors, and NO [21]. NO is a reactive metabolite synthesized by the NOS enzymes, produced in endothelial cells (eNOS) from l‐arginine. Its synthesis, calcium‐dependent, acts in cellular signaling and the control of cardiovascular functions. The action of NO is mediated by sGC, which, when activated in smooth muscle cells, increases cGMP concentration, resulting in vasodilation and relaxation of vascular smooth muscle cells (VSMCs) [22]. Therefore, the actions of boldine on these pathways were investigated.

**FIGURE 2:**
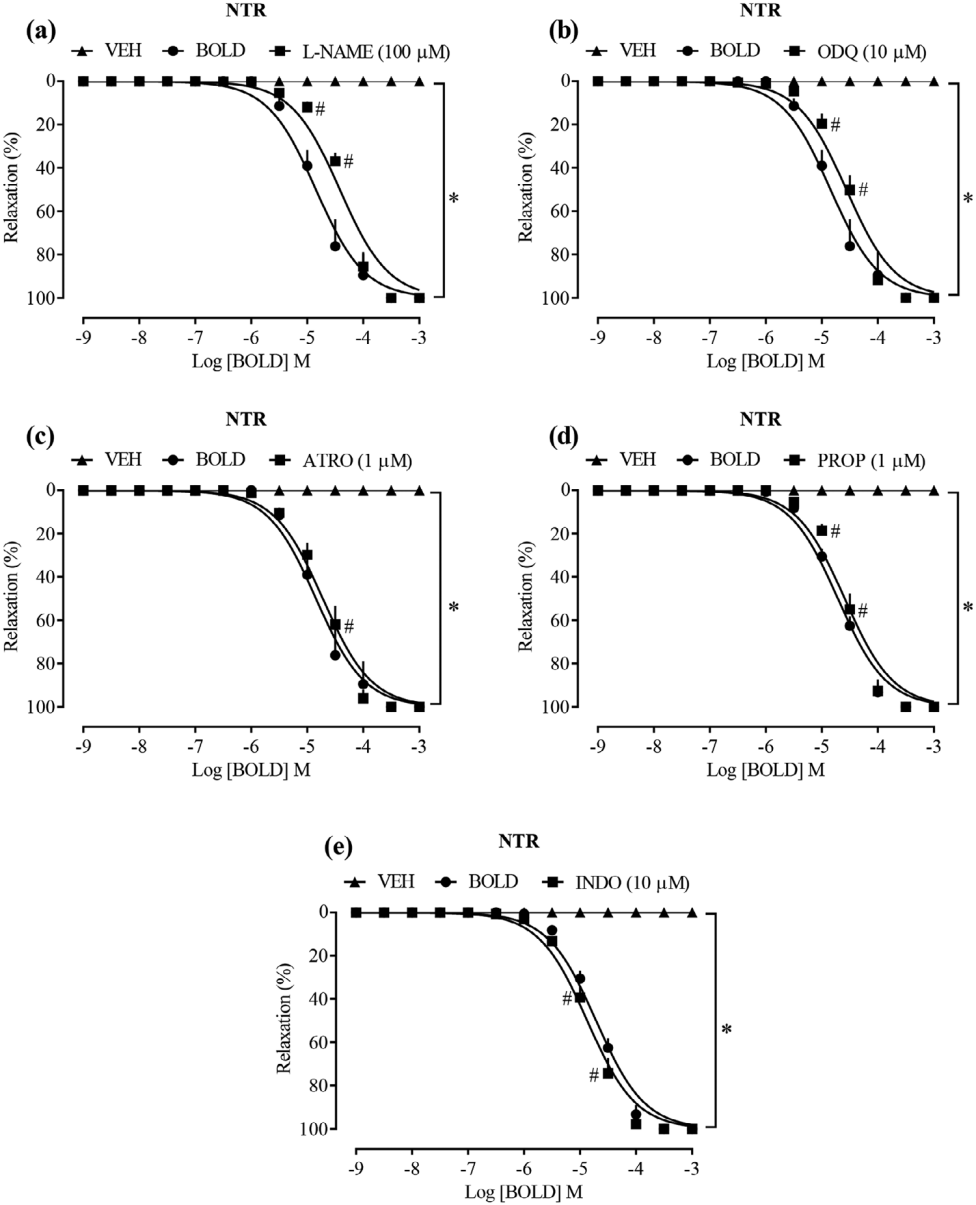
Endothelium‐independent relaxation of boldine on aortic rings of NTR. Effect of boldine in the presence of l‐NAME (a), ODQ (b), ATRO (c), PROP (d), and INDO (e). Statistical analyses were performed using a two‐way analysis of variance followed by Bonferroni's multiple comparison test. ∗*p *< 0.05 when compared to the vehicle group and #*p *< 0.05 when compared to the boldine group. l‐NAME, *N*ω‐nitro‐l‐arginine methyl ester; NTR, normotensive rat; PROP, propranolol.

When l‐name (100 µM), a nonselective inhibitor of NOS enzyme, as well as ODQ (10 µM), an inhibitor of the GCs enzyme, were previously added in concentrations already elucidated to prevent ACh‐induced relaxation, these inhibitors were unable to significantly interfere with the relaxation observed by the addition of boldine in aortic rings previously contracted with Phe (Figure 2a,b), LogEC50 −4.418 and −4.563, respectively. Although the data obtained in the relaxation curve show a slight shift to the right between the groups, its final response was not different, suggesting that the NO/GCs/cGMP pathway appears to have minor relevance for the relaxing effects of boldine.

G protein‐coupled receptors (GPCR) located on the intracellular surface of the membrane respond to ligands through the activation of Gα proteins (Gqα, Giα, and Gsα), which have different roles in vascular signaling. Gqα activates phospholipase C (PLC) to increase IP_3_ and DAG production, whereas Giα has the opposite effect. In the endothelium, Gqα‐coupled muscarinic receptors can be blocked by ATR. β‐Adrenergic receptors associated with Gsα activate adenylyl cyclase (AC) to increase cAMP, involved in vasodilation, and can be blocked by PROP in vascular muscle cells [23, 24, 25]. To analyze the participation of prostanoids, Ind, a nonselective inhibitor of cyclooxygenase, was used to block the production of prostanoids such as prostacyclin (PGI_2_). PGI_2_, produced in the endothelium, acts on GPCR in VSMCs, increasing cAMP and consequently promoting vascular smooth muscle relaxation by preventing actin‐myosin interaction [26, 27]. However, the vasorelaxant effect provided by boldine was not modified in the presence of any of these blockers (Figure 2c–e), LogEC50 −4.418, LogEC50 −4.563, and LogEC50 −4.873, respectively, suggesting that the effect of the compound does not depend on activation of these routes.

Potassium (K^+^) channels are crucial in controlling vascular tone, as they regulate membrane potential through K^+^ efflux, causing membrane hyperpolarization. This leads to the closure of voltage‐dependent Ca^2+^ channels and, consequently, the relaxation of smooth muscle [28, 29]. In endothelial cells and arterial smooth muscle, several subtypes of potassium channels have been identified, each with specific functions in vascular physiology. These channels can be broadly categorized into calcium‐activated (K_Ca_), sodium‐activated (K_Na_), voltage‐gated (K_V_), inwardly rectifying (K_IR_), and two‐pore domain (K_2P_) potassium channels. The complex interplay of these potassium channels subtypes is essential for precisely controlling vascular tone and, consequently, regulating blood pressure [30]. When investigating the involvement of K^+^ channels, TEA was incubated at a concentration of 1 mM, which acts as a blocker of K^+^ channels activated by Ca^2+^, capable of selectively inhibiting subgroups 2.1, 2.2, and 2.3, whereas at a concentration of 10 mM, it acts as a nonselective blocker of K^+^ channels that inhibits subgroups 2.3 and 3.1.

**FIGURE 3:**
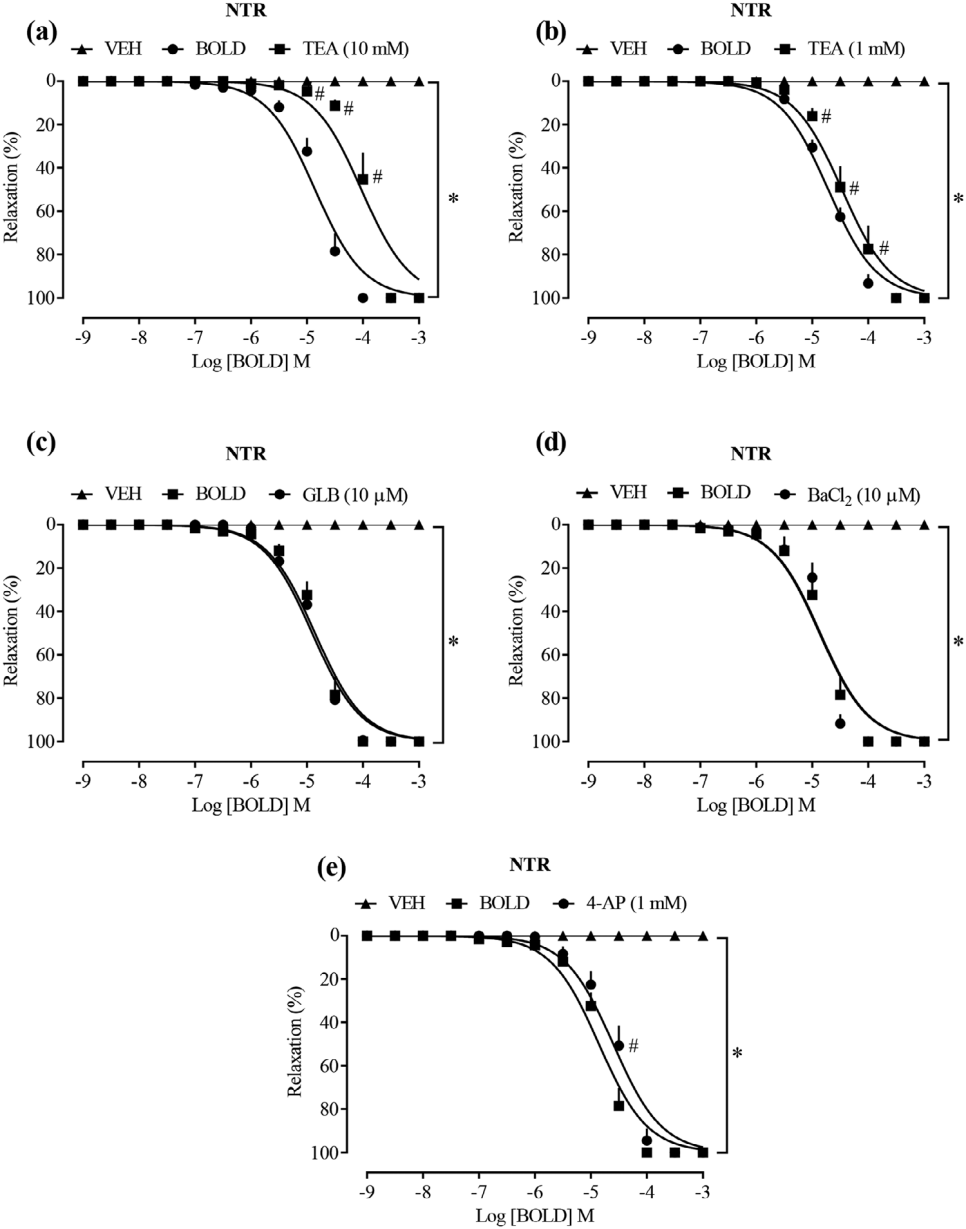
Role of K^+^ channels in the relaxant effect induced by boldine. Effect of boldine in the presence of TEA 10 mM (a), TEA 1 mM (b), GLB (c), 4‐AP (d), and BaCl_2_ (e). Statistical analyses were performed using a two‐way analysis of variance followed by Bonferroni's multiple comparison test. ∗*p *< 0.05 when compared to the vehicle group and #*p *< 0.05 when compared to the boldine group. NTR, normotensive rat; TEA, tetraethylammonium.

TEA at a concentration of 10 mM (Figure 3a), LogEC50 −4.029, which acts as a nonselective blocker of K^+^ channels, had a significant shift of the curve to the right, whereas at a concentration of 1 mM (Figure 3b), LogEC50 −4.470 the detachment was slightly significant, suggesting a reduction in the relaxing effect of the compound; however, at both concentrations, there was no interference in the maximum effect. In the presence of glibenclamide (GLB), selective blocker of K_IR_ 6.1 and 6.2 also known as ATP‐sensitive inwardly rectifying K^+^ channels (Figure 3c), LogEC50 −4.907, and barium chloride (BaCl_2_), nonselective blocker of K_IR_ channels (Figure 3d), LogEC50 −4.871, these were not able to significantly alter the vasorelaxant activity of the compound. On the other hand, a shift of the relaxation curve to the right of boldine was observed in the presence of 4‐AP, a selective blocker of K_V_ channels (Figure 3e), LogEC50 −4.601, suggesting that, at least in part, these channels may contribute to the relaxing effects of boldine.

On the basis of these data, the role of Ca^2+^ channels in the actions of boldine was investigated, which regulate vascular tone and the contraction of VSMCs. The elevation of Ca^2+^ concentration occurs through the release of Ca^2+^ from the sarcoplasmic reticulum (SR) or through the influx of extracellular Ca^2+^ through l‐type voltage‐operated calcium channels (VOCCs), activated by agonist receptors and/or membrane depolarization. The Ca^2+/^calmodulin complex activates myosin light chain kinase (MLCK), phosphorylating myosin and allowing its interaction with actin, resulting in the contraction of vascular smooth muscle [31]. The vasoconstriction mechanism induced by agonists involves the production of second messengers such as inositol triphosphate (IP_3_) and diacylglycerol (DAG). For example, Phe is a selective agonist of α1‐adrenergic receptors, which are coupled to the G_q_ protein. When activated, they lead to the production of IP_3_ and DAG through the activation of phospholipase C. IP_3_ acts on receptors in the SR to promote the release of Ca^2+^, whereas DAG activates protein kinase C (PKC) [32]. Vascular smooth muscle relaxation requires a reduction in intracellular Ca^2+^ concentration and the dephosphorylation of myosin by myosin light chain phosphatase.

**FIGURE 4:**
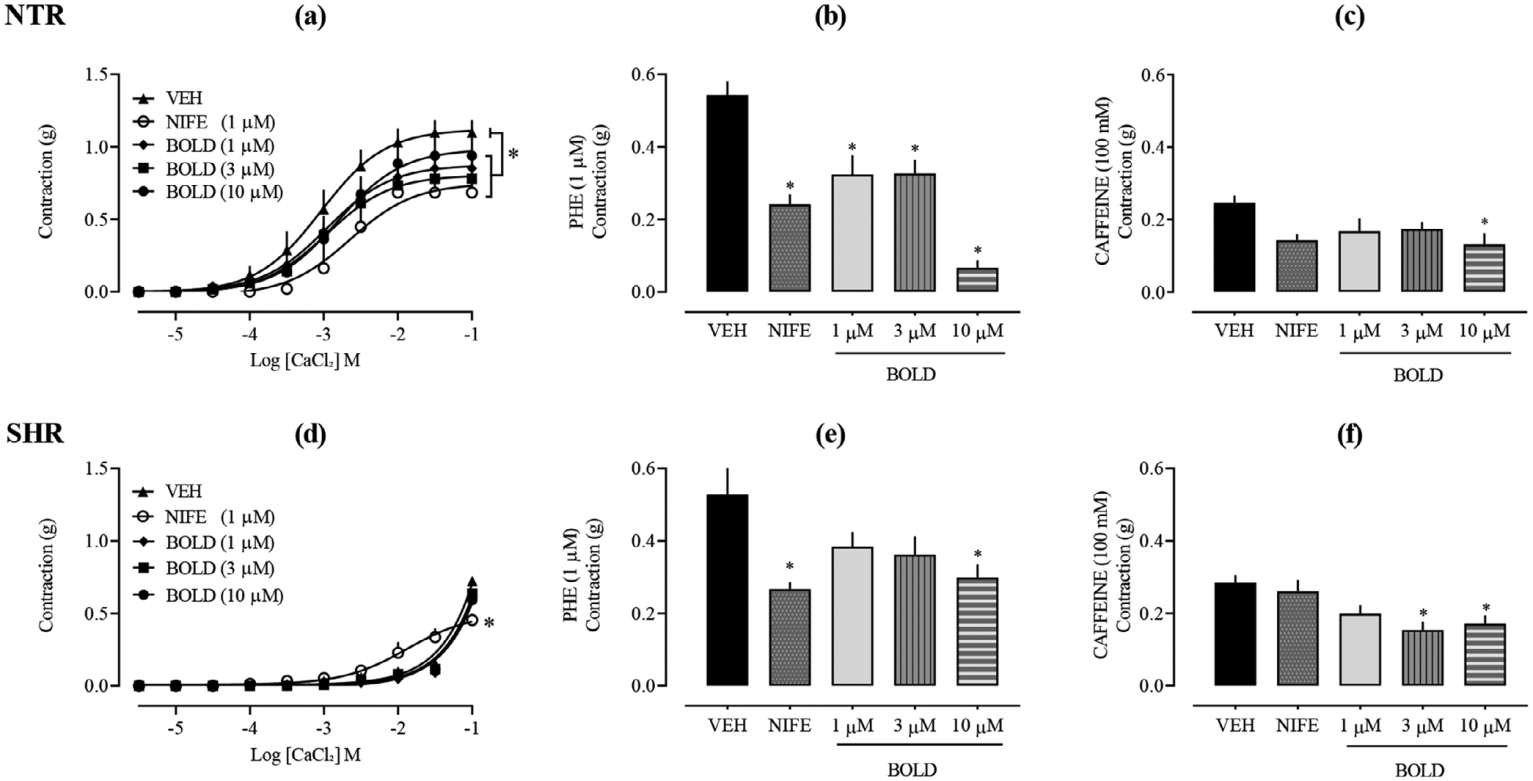
Role of Ca^2+^ channels in the vascular effects of boldine. Concentration–response curves to CaCl_2_ in the presence or absence of boldine: 1, 3, and 10 µg/mL in aortic rings from NTR and SHR (a and d, respectively). Contraction induced by Phe (b and e) and caffeine (c and f) in the presence of boldine in calcium‐free PSS. Nifedipine (NIFE—1 µM) was used as a positive control. Statistical analyses were performed using a two‐way analysis of variance followed by Bonferroni's multiple comparison test. ∗*p *< 0.05 when compared to the vehicle group. NTR, normotensive rat; Phe, phenylephrine; SHR, spontaneously hypertensive rat.

Initially, the role of transmembrane Ca^2+^ channels was investigated. When boldine was incubated at concentrations of 1, 3, and 10 µg/mL in a calcium‐free depolarizing PSS, it significantly reduced the contractile response to CaCl_2_ in aortas from NTR (Figure 4a), indicating that boldine reduces the influx of Ca^2+^ from the extracellular environment to the cytosol. As a positive control, NIFE, a commonly prescribed antihypertensive and antianginal drug, was used. NIFE inhibits the entry of calcium ions by blocking l‐type VOCC in vascular smooth muscle and myocardial cells. The reduction in intracellular Ca^2+^ decreases peripheral arterial vascular resistance and dilates coronary arteries, leading to a reduction in systemic blood pressure and an increase in oxygen supply to the myocardium [33].

In sequence, the calcium channels located in the SR were investigated. For this, two constricting agents (Phe and caffeine) were used, which were exposed to the aortic rings previously kept in a free Ca^2+^ solution, which prevents its entry from the extracellular environment. Phe, upon binding to the α1‐adrenergic receptor, triggers the formation of IP_3_. This IP_3_ then binds to its receptors on the SR membrane, causing the release of Ca^2+^ ions into the cytosol [34, 35]. Conversely, caffeine interacts with RyRs receptors, also known as ryanodine receptors (named after the plant‐derived alkaloid). This interaction also causes the release of Ca^2+^ from reticular stores. As shown in Figure 4b,c, the prior addition of boldine at concentrations of 1, 3, and 10 µg/mL in Ca^2+^‐free PSS inhibited the contractile response induced by Phe. When aortic contraction was induced by caffeine, inhibition of the contractile response occurred only at the 10 µg/mL concentration of boldine. This suggests that the IP_3_ receptors and RyRs receptors located in the SR, at least partially, participate in the modulation of vascular tone promoted by boldine.

Supporting the findings, numerous vasoactive alkaloids reviewed in the literature share an isoquinoline structure and have demonstrated the ability to induce endothelium‐independent vasorelaxation by blocking Ca^2+^ entry into VSMCs and causing aortic relaxation. The mechanism of action of isoquinoline alkaloids appears to involve the blockade of l‐type VOCCs in VSMCs [36]. Considering that the relaxing effect of boldine in NTR showed a significant relationship with both endoplasmic reticulum‐based IP_3_ and RyRs and transmembrane Ca^2+^ channels, the effects of boldine on aortas from SHR were also evaluated. However, in SHR vessels, boldine was unable to reduce the contractile response induced by CaCl_2_ (Figure 4d). Considering that the literature describes alterations in calcium‐mediated signaling in SHR, it is possible that these aortas exhibit impairments in this pathway, either due to changes in receptors and/or channels or the intracellular signaling cascade. Nonetheless, as boldine also demonstrates a vasorelaxant effect in the aorta of SHR, it suggests that transmembrane Ca^2+^ channels are not essential for the observed effect. On the other hand, when boldine was incubated at a concentration of 10 µg/mL in calcium‐free PPS, it significantly reduced the contractile response to Phe by 43.33%, as well as reducing caffeine‐induced aortic contraction by 39.65%, compared to the vehicle. This confirms the involvement of endoplasmic reticulum‐based IP_3_ and RyRs in the vascular actions of boldine also in hypertensive aortas.

This study demonstrated for the first time that the alkaloid boldine has an endothelium‐independent vasorelaxant effect, suggesting that the compound crosses the endothelium and directly evokes a vascular response in the smooth muscle. The results further indicated that the underlying mechanisms may involve reductions in cytosolic Ca^2+^ concentration, likely through the blockade of VOCC or inhibition of IP_3_ and RyRs receptors. The isolated aorta model has proven to be a valuable tool for validating vasoactive properties and provides a useful pharmacological tool for in vitro analysis. This is due to the low number of animals required, good reproducibility of experiments, and the ease with which results can be extrapolated to in vivo models. This model helps the analysis of bioactive compounds derived from plants used in traditional medicine to treat CVDs such as hypertension [37]. However, despite the significant results described here, further studies are needed to deeply elucidate the mechanisms responsible for the vasorelaxant effect, as well as the compound's effects on other systems and organs that contribute to the control of vascular tone and blood pressure.

## 4 Conclusion

The results presented here reveal that boldine causes endothelium‐independent vasorelaxation in aortic rings from NTR and SHR, supporting its involvement in controlling vascular tone. Its mechanism of action appears to be mainly involved with endoplasmic reticulum‐based IP3 and RyRs and transmembrane calcium channels in the aortas of NTRs. In aortas from hypertensive rats, however, the vascular actions of boldine were confirmed exclusively through the involvement of IP_3_ and RyRs receptors. There is also a discreet participation of pathways modulated by NO and K^+^ channels, which collectively may contribute to the actions observed here.

## Author Contributions

Martina Odebrecht Cavichiolo and Rita de Cássia Vilhena da Silva performed the experiments. Martina Odebrecht Cavichiolo, Rita de Cássia Vilhena da Silva, and Priscila de Souza analyzed the data. Martina Odebrecht Cavichiolo wrote this article. Priscila de Souza and Valdir Cechinel Filho have designed and supervised this study. Priscila de Souza corrected and edited the final version of the manuscript. All authors have reviewed and approved the final version of the manuscript for publication.

## Ethics Statement

All methodologies and procedures described here followed the experimental protocols previously approved by the Ethics Committee for the Use of Animals at UNIVALI (protocol no. 001/23) and were conducted under all established ethical standards.

## Conflicts of Interest

The authors declare no conflicts of interest.

## Data Availability

The data that support the findings of this study are available from the corresponding author upon reasonable request.
